# Patient engagement with consumer wearable devices in the electronic health record

**DOI:** 10.3389/fdgth.2026.1784621

**Published:** 2026-05-07

**Authors:** Michael Sobolev, Allistair Clark, Mitchell Kamrava, Joshua Pevnick, Brennan Spiegel, Raymond Duncan, Gillian Gresham

**Affiliations:** 1 Department of Medicine, Cedars Sinai Medical Center, Los Angeles, CA, United States; 2 Center for Health Policy and Economics, University of Southern California, Los Angeles, CA, United States

**Keywords:** internet of things (IoT), mobile health, personal health, remote patient monitoring, survival analysis

## Abstract

Data from wearable devices have the potential to transform personal health, clinical care and biomedical research. The purpose of this study was to quantify early adoption and short-term engagement with consumer-based wearable devices connected to an EHR outside of a study setting. We analyzed device data from 843 patients (mean age 48 years, 41% female) who connected their devices to the electronic health record (EHR) of a large academic medical center in the US between 2023 and 2025. The most popular connected consumer devices were the Apple Watch (56%), followed by the Oura ring (7.7%), and the Fitbit activity tracker (4.6%). Using survival analysis, we found that 692 (82%) patients remained engaged at three months, with actively connected devices. Cox regression models identified statistically significant differences in time to disengagement based on gender and step count. Regular and updated analyses of device adoption and engagement patterns are therefore warranted to increase the value of these devices and their data for the purpose of health monitoring and improvement.

## 1 Introduction

Consumer wearable devices enable real-time collection of activity and health data, providing transformative opportunities for monitoring, diagnosing, and managing a wide range of health conditions (1, 2). The emergence of advanced wearable technology including the introduction of smart watches, around 2015, marked a shift towards multifunctional devices that integrate communication, apps, and health data. While basic step trackers (e.g., pedometers) and research-grade accelerometers (e.g., ActiGraph) have traditionally been utilized in physical activity research, there has been a growing trend toward the use of participants' own personal smartwatches both within and beyond the research setting. These devices are increasingly ubiquitous and preferred by users, not only as research tools, but also as communication devices, fashion accessories, and means to monitor their own health through features such as heart rate monitoring, notifications, and integration with smartphones and health apps (3–6). Some of the most common devices sold in the USA (e.g., Apple Watch) include multifunctional health platforms offering real-time feedback, gamified fitness goals, social competition, and tailored health insights—fostering sustained user interaction and daily use (7–9). Consequently, an increasing proportion of adults residing in the United States (US) began to use wearable devices daily and expressed willingness to share data with healthcare providers (10, 11). Thus, the evolving landscape of consumer wearable devices requires frequent and continued evaluation of patient adoption and engagement.

As early as 2015, healthcare systems have also been integrating wearable devices and their data into the electronic health record (EHR) and patient portals (12). Despite this availability, numerous barriers to the implementation of wearable health technology exist and patient engagement has previously been a key challenge (6), with some early studies indicating early disengagement post adoption, with about half of the users reporting that they stopped using the device after six months, a phenomenon termed the law of attrition (13, 14). However, user engagement has increased over the past decade as a result of evolving technology, personalized features, continuous health monitoring, and seamless integration with smartphones (15–18). Therefore, digital health research has been increasingly focusing on various elements of engagement, from adoption to continuous use (19, 20).

Most prior research examined patient engagement with wearables as part of a clinical trial and focused on a single device and purpose (19). There remains a need to investigate objective engagement with wearable devices in healthcare settings, going beyond subjective self-report of usage (11) and engagement in controlled research settings, such as clinical trials (21). Within EHR-connected workflows, evidence regarding consumer-based wearable devices has been more limited and has largely emphasized initial uptake and feasibility. As such, fewer studies have focused on early, short-term engagement patterns following EHR connection Thus, the purpose of this study was: (a) to characterize recent patterns of patient adoption of consumer wearable and connected devices linked to the EHR in a large US healthcare system; (b) to quantify patient engagement with these devices over time, and (c) to identify patient-level predictors of early disengagement. Building on previous research studying engagement and attrition of use of wearable devices, we operationalized disengagement as the last recorded daily step count observation per patient (19). To expand on previous work and focus on recent trends in wearable device engagement, we examine new users who connected their devices to a health system's EHR in 2023 and 2025 and investigate time to disengagement over the following three-month period for each patient.

## 2 Method

### 2.1 Study design and participants

The study had two overarching aims: (1) to quantify recent patient adoption and engagement with consumer wearable and connected devices in a large US healthcare system (Los Angeles, CA) (01/01/2023-09/30/2025), including the distribution of linked device types and availability of valid activity data; and (2) to identify patient-level predictors of early disengagement, including demographic factors, early step count, and exploratory device-type differences in time to disengagement.

Our healthcare system began integrating wearable health data into the EHR in March, 2015 and has been allowing patients to connect their consumer-directed health wearable devices directly through the Epic MyChart-powered patient facing portal, thus allowing the passive collection of objective activity data (12, 22). We used this data to retrospectively analyze device data, defined as any connected consumer-based device (e.g., wearable activity tracker, phone, phone-based application) used to track activity, connected to the EHR between January 1, 2023, to December 31, 2025, where engagement was defined as the presence of both step counts and heart rate data (See Supplementary File S1 for additional details. This analysis was intentionally restricted to recent new adopters (2023−2024) to examine early disengagement within 3 months of initial device connection. Accordingly, the cohort was not designed to represent all patients who have ever linked a wearable device to the EHR, including early adopters and long-term users. Rather, the present study addresses a complementary question to our prior 2015–2021 analysis (23) with 1-year follow-up, which examined longer-term engagement patterns. This study included all patients from the database who were 18 years of age or older and had any wearable health device connected to EHR during the study period, and did not have a previously connected device. This investigation obtained ethics approval through the Cedars-Sinai IRB (IRB ID: STUDY00002591) for which a waiver of consent was granted due to the minimal risk to patients and absence of direct subject interaction.

### 2.2 Variables

The extracted dataset contained 61 variables in total. The present analyses used a study-specific subset of 10 variables relevant to the objectives, including patient demographics (e.g., age, gender, race, ethnicity), wearable device metadata (e.g., device type and date of first device linkage), and wearable measurement fields (e.g., measurement type, measurement value, date recorded on-device, and date uploaded to the EHR). The Supplementary File S1 includes a complete list of the analytic variables used in this study. To quantify engagement with wearable devices over three months, we performed a series of survival analyses. We selected three months of follow up to investigate the short-term engagement patterns of participants who are new adopters of wearable technologies. Furthermore, setting a relatively early period of follow up has been supported by other literature which indicate that early engagement patterns predict long-term engagement (24, 25). This window captures the period when disengagement is most likely (18). Thus, by limiting the sample to new adopters and focusing on a 3 months timespan, we aim to more clearly identify factors that predict early disengagement. More information about the data preparation process can be found in the Supplementary Material. For these survival analyses, we defined an event as the patient's last daily step count reported (i.e., the participant's last observation date), thus indicating device disengagement from the EHR framework.

### 2.3 Statistical analysis

To identify and quantify the types of wearable devices over the 2023–2025 period, we conducted a descriptive analysis of device and patient characteristics and compared across device types. For all analyses, we defined the follow-up period as 3 months from the patients' first observation. Therefore, only patients who had connected their devices on or before September 30th, 2025, were included in the analysis to allow for sufficient follow-up time to the end of the year. Patients whose last observation occurred before their respective 3-month endpoint (e.g., if they connected a device on September 30th 2025, then their corresponding 3-month endpoint would be December 30th, 2025) were considered to have had their device(s) disengaged from the system and thus marked as experiencing the event. Total count and proportion of devices that remained connected at the one-year mark are reported. Kaplan–Meier survival curves were generated to evaluate connected wearable device engagement by demographic variables including gender and the top four most types of devices that were connected to the EHR. To investigate and define longitudinal relationships between patient engagement and sociodemographic variables, we then included all discussed variables as covariates in a multivariable Cox proportional hazards model to determine which factors significantly impacted device disengagement within this observational dataset. No *a priori* power calculations were conducted due to the retrospective, descriptive nature of the dataset and its sample specification of the entirety of the adult population which connected their wearable device(s) to the EHR workflow during the examined period. All analyses were applied through RStudio 4.2.3 (Posit Software, Boston, MA, USA), using the “survival” and “survminer” packages. The proportional hazards assumption for Cox regression was assessed for this model using the cox.zph function from the *survival* R package and found to be nonsignificant.

## 3 Results

### 3.1 Patient demographics

Of the 3,930 patients with observations present in the timeframe, the final dataset consisted of 843 eligible patients with connected devices before 9/30/2025, with a mean age of 48.2 (SD 15). Of the 843 final patients, 493 (58.48%) identified as male, and 506 (60.02%) patients were white (Table 1). The observations in the refined dataset used for this investigation consisted of daily step counts, which were the primary device data of interest. This metric was chosen due to its interpretability and its ubiquitous presence, consisting of half of all observations in the original dataset.

**Table 1:** Demographics of patients who connected a device between 2023 and 2025.

Total: *N* = 843		
Variable	Count *n*	Percent *%*
Gender		
Male	493	58.48
Female	350	41.52
Race		
White	506	60.02
Black or African American	67	7.95
Asian	93	11.03
Other	114	13.52
Unknown	63	7.47
Ethnicity		
Hispanic	135	16.01
Non-Hispanic	624	74.02
Unknown	84	9.96
	***Mean (SD)***	***Interquartile range***
Age	48 (14.91)	36–58 (22)
Steps	6,813 (4,462.78)	3,595–9,064 (5,469)

Initial identification of connected devices revealed a total of 1,181 unique devices with three primary data API sources: Apple Health Kit, Fitbit, and Google Fit. Across the refined dataset, the two most prevalent wearable devices connected by patients were Apple and Oura devices followed by Fitbit. While Fitbit data sources upload only observations from their respective devices, Apple HealthKit has the functionality to connect and aggregate objective health metrics from multiple devices and applications at once from multiple brand types. We explored the composition of device types under the Apple Health Kit umbrella by investigating the API source of each observation.

### 3.2 Device data

**Figure 1:**
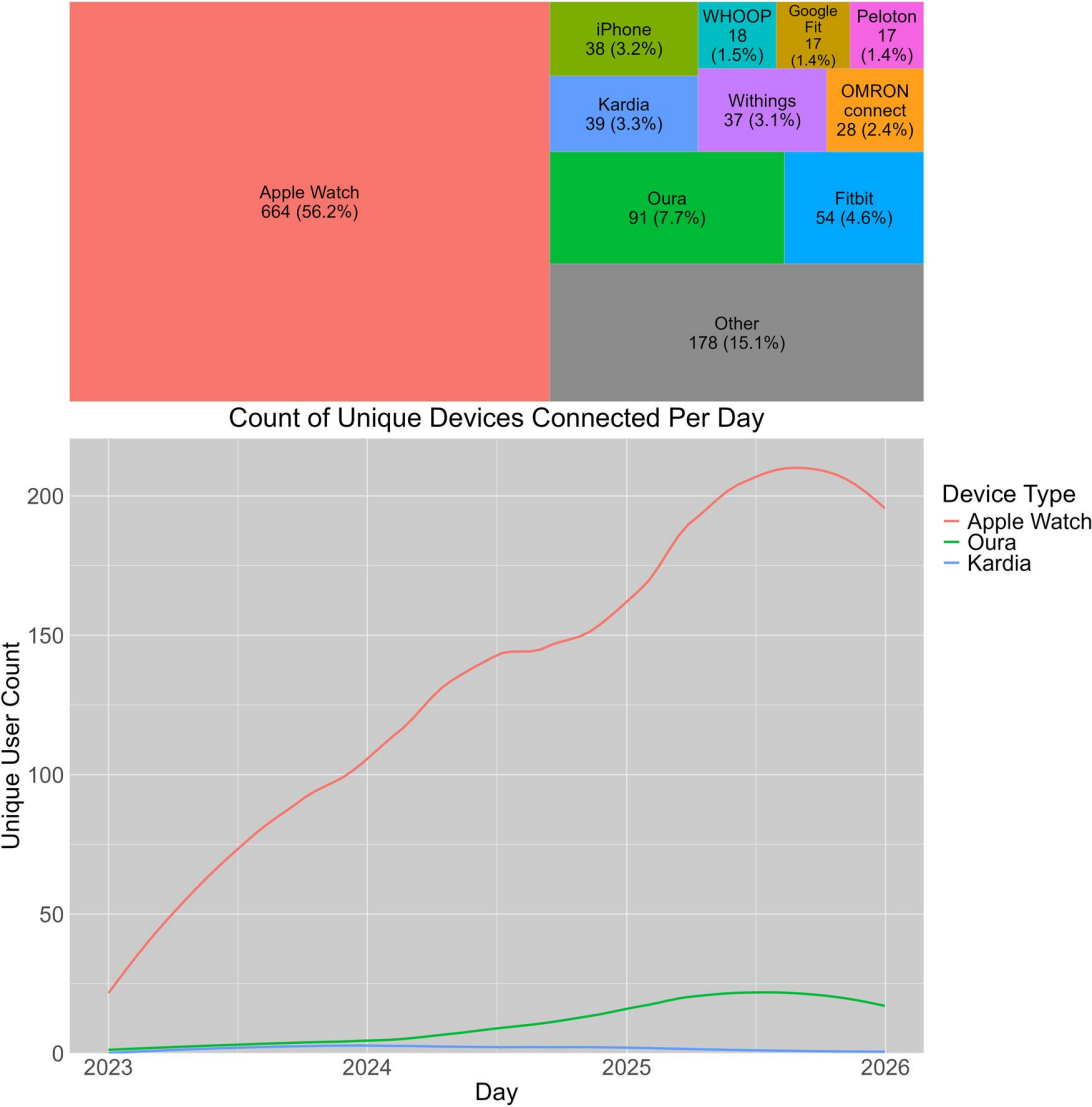
Treemap plot of devices type adoption and daily count of unique devices over time.

Included devices in this analysis were largely classed into 4 categories: On-phone applications that utilize the phone hardware to record health metrics (e.g., using the light and camera for pulse estimation, using the phone accelerometer and gyroscope to track steps), devices that are the same brand as their respective applications (e.g., Fitbit devices for the Fitbit app, Apple devices for Apple Health Kit, etc.) standalone blood pressure cuffs, and devices that have had their data aggregated and uploaded to the EHR through another brand's application. As an example of this latter category, Apple Health Kit was found to have consisted of 50 distinct device brands (e.g., device brands such as Oura, Peloton, Withings, Kardia, etc.) across 1,072 separate devices apart from Fitbit and Google Fit devices. See Figure 1 for full breakdown of devices connected to the workflow.

To further investigate the proportion of total devices by type, we then parsed the count of unique devices connected per month across the entire date range, spanning from 01 to 01-2023 to 12-31-2025 (see Figure 1). Visualizing this count supports what we observed from raw counts of device types. Specifically, Apple Health Kit-type devices were the most prevalent device type across the two years with a steadily increasing proportion of new devices being connected to the EHR workflow. In contrast, both Fitbit and Google Fit API connections saw relatively little growth in number of devices connected and furthermore significantly fall off past November of 2024.

### 3.3 Patient engagement

**Figure 2:**
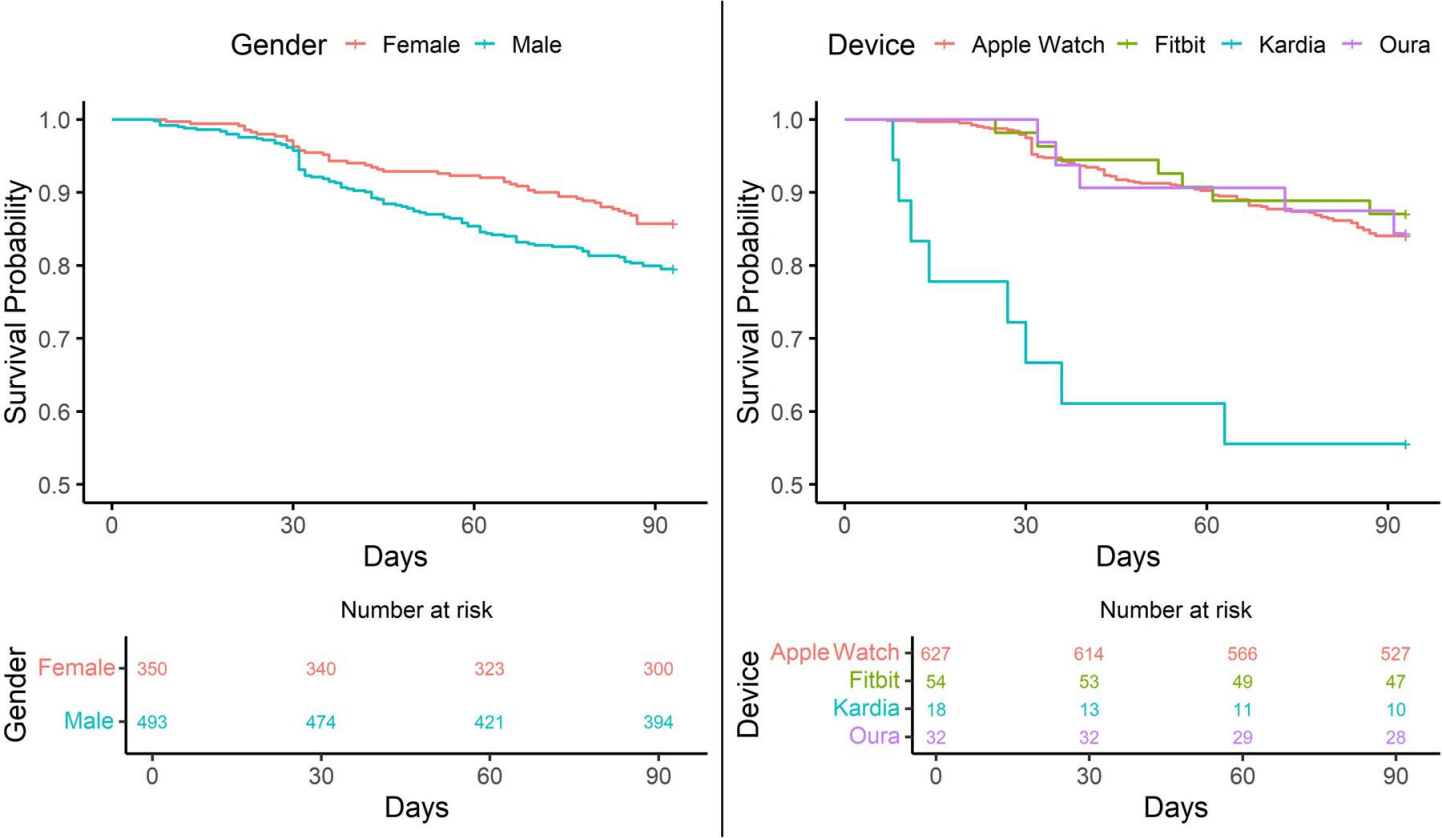
Time to device disengagement by gender and device type over a period of three months.

Kaplan–Meier survival curves estimating patients' probability of disengagement with the EHR system across a 3-month period from patients' initial day of device connection are depicted in Figure 2. Log-rank results for these Kaplan–Meier curves indicate a significant difference in probability to disconnect from the EHR by gender (*p* = .02) but no difference in probability to disconnect by device type (*p* = 0.9).

Overall, of the 843 patients included in the survival analyses, 692 (82.09%) patients remained engaged with the EHR system beyond the 3 Month follow up period. As such, median survival (reaching 50% disengagement) was not reached within 3 months for this sample when conducting Kaplan–Meier survival analyses. Figure 1 in the Supplementary Material depicts the overall, ungrouped survival curve.

To further explore factors that impact patient engagement over time, we fit a Cox proportional hazards model including key sociodemographic variables with objective measurement data collected from patient wearables as covariates. Table 2 contains the entire set of variables, including age, gender, and median step count within the first week of device connection. Patients who identified as male had a statistically significant higher likelihood to disengage than patients who identified as female by 1.52 times the hazard. The age quartiles covariate, with ages 18–34 as reference, was not statistically significant. Finally, median step counts from the first 7 days of observations per patient were entered into the model, and categorized by less than 5,000 steps, 5,000–7,500 steps, 7,500–10,000 steps, and greater than 10,000 steps. Results indicate that patients who took between 5,000 and 7,500 during their first week of engagement were at a significantly higher risk of disengagement compared to the groups that took 7,500–10,000 steps.

**Table 2:** Multivariable cox proportional hazards models for time to device disengagement adjusted for age, gender, race, ethnicity and baseline week median step counts.

	Coefficients		
Covariates	Hazard Ratio (HR)	95% Confidence Interval	*p*-value
Age Group (Ref: 18–34)			
35–44	1.04	0.65–1.69	0.860
45–55	0.89	0.53–1.49	0.650
56–100	1.22	0.78–1.93	0.386
Gender (ref: Female)			
Male	1.52	1.08–2.14	0.016
Step Count Categories (ref:5,000–7,499)			
<5,000	0.83	0.57–1.20	0.324
7,500–10,000	0.59	0.35–0.99	0.046
>10,000	0.77	0.46–1.31	0.342

## 4 Discussion

The current study evaluated recent trends in consumer wearable devices adoption and engagement by analyzing consumer-directed health devices linked by patients to an EHR within a large academic medical center in the US. We used passively collected data from patients who connected their devices voluntarily to the EHR using a patient portal. The most connected device during the study period was the Apple Watch (56%), followed by the Oura ring (7.7%) and the Fitbit activity tracker (4.6%). We observed a high level of continued device engagement at three months with approximately 82% of users still engaged 3 months after connecting. In a separate analysis of the same data source from 2015 to 2022, we found similar engagement levels (23). This suggests that this pattern is stable over time and not changing over time or due to unique characteristics of the current sample. We also found that disengagement mostly emerges at around one month after device connection (24), which can provide a direction to guide future research concerning the dynamic of disengagement. This paper provides an additional contribution to the effort of analyzing engagement patterns to evaluate the feasibility of using connected devices and data for personal health, clinical practice, and biomedical research. Moreover, due the changing landscape of wearable devices, research on engagement must be continuously re-examined (26).

Further, we examined factors that could predict early disengagement with wearable devices through a series of time-to-event analyses. We found that patients who identified as male had 1.52 times the hazard to disengage than patients who identified as female. This finding, though from a non-research-based cohort, is consistent with previous findings that indicated higher likelihood for women to enroll and engage with biomedical and mobile health research, which may imply that this rate of disengagement exists beyond incentivized research conditions (17). We also examined step count as a risk factor for disengagement, the most prominent indicator of physical activity and a digital biomarker of various health outcomes (27). We found that patients with a baseline step count between 5,000–7,500, which represent the most average category of physical activity, are more likely to disengage than patients with a slightly higher than average step count of 7,500–10,000.

Although there could be multiple explanations for these findings, theories of innovation and technology acceptance (28) predict that demographics factors such as gender will become less prominent predictors of engagement when technology is widely adopted. Similarly, prior research demonstrated that those with higher physical activity levels were more likely to adopt and engage with the early wearable devices (13), but this effect is predicted to attenuate in recent years as wearable devices gain popularity. On a general note, our analysis takes place a decade since the release of the Apple Watch, and lends evidence of the ubiquity of Apple devices in the consumer health marketplace in the US (29), with 80% of devices using HealthKit and 60% of all devices connected within our sample were Apple Watches. At the same time, emerging devices like the Oura ring are gaining popularity, especially for sleep tracking, which is evident in our data as well (30).

This study provides insight into recent patterns of patient engagement with personal consumer-based wearable devices, in terms of connection to the EHR. Our findings suggest that short-term engagement levels are high, and that connected devices could be further leveraged for future clinical care and research. Unique to this analysis was our ability to analyze multiple devices at the same time from the EHR, and without relying on data from a clinical trial that choose a specific device for their trial, such as Fitbit device (19). Nevertheless, our findings could deviate from prior results due to the relatively short-term follow-up period that was examined and the unique setting and population. Further, our analysis was limited to the data available and connected devices in this particular cohort, thus may not be representative of other health system EHRs. An important consideration is that this cohort consisted of patients who voluntarily chose to use and connect a wearable to our system, which may represent a subgroup that differs meaningfully from the broader health system population. Future analyses will focus on long-term engagement patterns, and are likely to yield different results, especially considering the addition of other unaccounted for risk factors (e.g., disease status, cancer diagnosis, etc.) (19, 31). Step-count analyses are limited by measurement heterogeneity across manufacturers, platforms, and device generations as well as potential connect through third-party applications that do not reliably export data linked to the EHR. In addition, because step-detection algorithms are not standardized and raw accelerometer data were unavailable, associations between step count and disengagement should be interpreted cautiously, particularly across device types. In conclusion, continuous efforts to understand and encourage device integration and engagement will increase the clinical and research value of these devices.

## Data Availability

The de-identified, aggregate data supporting the conclusions of this article will be made available by the authors upon request and necessary institutional data use agreements have been formalized.
