# Supplementary material for: Patient engagement with consumer wearable devices in the electronic health record
Source: Front Digit Health. 2026 May 7;8:1784621. doi: 10.3389/fdgth.2026.1784621 (PMC13189792; doi:10.3389/fdgth.2026.1784621)

Supplementary File

1.1 Supplemental

Overall data pipeline

All records reviewed in this study originated from wearable health device (WHD) data extracted from the CSLink reporting environment. The overall framework for WHD data collection was as follows: patients linked supported consumer health platforms to MyChart; patient-generated data were ingested into the Epic transactional electronic medical record (EMR) through proprietary interfaces; WHD data were stored separately from clinician-entered data and made available for clinical review in a dedicated flowsheet; and transactional EMR data were exported once daily to a reporting environment, from which longitudinal WHD data were extracted for analysis.

New users of the MyChart CSLink portal were prompted at first sign-in with an instructional page describing how to link WHDs to CSLink.

Platform-specific linkage pathways

For Fitbit and Withings, users typically initiated linkage through the web-based MyChart interface. After logging into MyChart, the user selected Link Account and then selected the relevant Connect option, which redirected the user to the vendor login page, such as Fitbit or Withings, to authenticate and authorize account linkage. Once authorization was completed, a direct connection was established between the vendor cloud and the Epic transactional system. In these cases, data transmission to Epic occurred through the vendor-cloud-to-Epic interface rather than through the vendor mobile app or the MyChart mobile app, although the data could subsequently be viewed in the MyChart mobile application.

Apple Health linkage was initiated through the MyChart mobile application rather than web MyChart. Within the MyChart mobile workflow, users could connect Apple Health and specify which data types were shared with MyChart. Apple Health may also receive data from third-party apps and devices, such as Omron and others, and when such connections were enabled, those data could pass through Apple Health to MyChart. Accordingly, Apple Health or HealthKit can function as an aggregation layer for multiple upstream sources.

Google Fit data were also ingested through a proprietary Epic interface; however, the implementation workflow was not the focus of the present analysis.

Source identification and interpretation

Because Apple Health or HealthKit may aggregate data from multiple upstream inputs, including smartphone sensors, smartwatch sensors, and third-party apps or devices, records associated with Apple Health or HealthKit were treated as a platform-linked source category and should not be assumed to reflect a single hardware source.

More generally, this study distinguishes source or platform linkage in the EHR data pipeline from definitive hardware-level provenance for each individual measurement. Accordingly, device-source labels available in the reporting environment were interpreted as indicators of the linked platform or

source category captured by the EHR pipeline, rather than as definitive confirmation of the exact hardware that generated each observation.

Handling of overlapping inputs

Apple Health or HealthKit was recognized as a potential aggregation layer that may combine measurements originating from more than one upstream source. For that reason, Apple Health-linked records were not interpreted as representing a single device class in all cases.

In the study, overlapping inputs were addressed analytically by classifying records according to the source or platform information available within the EHR export and by explicitly acknowledging that platform-linked categories, particularly Apple Health or HealthKit, may reflect combined upstream sources. The analysis therefore distinguishes EHR-captured source attribution from definitive hardware-level provenance.

For analyses using daily step-count data, including survival models with step-count covariates, some patients had multiple same-day observations of the same metric, including cases potentially reflecting multi-device or platform aggregation, such as phone and smartwatch contributions within Apple Health-linked data. To avoid multiple entries per patient-day and reduce potential inflation from same-day duplicate or overlapping reports, step-count data were collapsed to a single daily patient-level value by taking the median of all valid same-day step observations for that patient, such that each patient contributed at most one step-count value per day in these analyses.

This distinction is important when interpreting step-count data. Records routed through Apple Health or HealthKit may reflect smartphone sensors, smartwatch sensors, third-party apps, or other connected devices, depending on the user's configuration. As a result, Apple Health or HealthKit records in this study should be interpreted as platform-linked observations rather than as unequivocal evidence of a single hardware source.

Data cleaning and analytic cohort derivation

Raw wearable-derived observations were first standardized by converting recorded dates to a uniform date format and separating records by measurement type.

Basic plausibility filters were then applied within each metric to remove clearly invalid or extreme values for pulse and step-count observations. Step-count data were further restricted by removing extreme high values and trimming the lowest 1% of step observations to reduce the influence of implausible or artifactual values. After value-level cleaning, exact duplicate records for the same person, date, device input, and measure were collapsed by replacing repeated values with the median within that duplicate set.

After record-level cleaning, participant eligibility criteria were applied to derive the analytic cohort. For the present analyses, we focused on adult participants with device-linked step-count data whose first qualifying step observation occurred between January 1, 2023 and September 30, 2025. Participants already represented in the earlier 2015-2022 connected-device dataset were excluded to limit overlap with prior cohorts and to estimate engagement in only recent, newly connected

patients, and participants whose first device connection occurred after September 30, 2025 were also excluded to preserve adequate potential follow-up time within the study window of 3 months. To improve confidence that retained observations reflected wearable-linked monitoring days rather than isolated uploads, participants were required to contribute at least 7 distinct verified wear days within the first 93 days after their initial qualifying observation. In the primary survival analyses, verified wear days were defined as days on which step-count and pulse observations were both available for the same participant on the same date. Participants were additionally required to have pulse data available, and demographic recoding procedures were then applied to harmonize race, ethnicity, age, and device-source categories for descriptive and time-to-disengagement analyses.

Variables

The analytic variables used in this study were: age, gender, race, ethnicity, API source, device name, date of first device linkage, measurement type (e.g., step count, pulse), measurement value, and date of observation.

API source refers to the external platform through which patient-generated health data were linked to the electronic health record, such as Apple HealthKit, Fitbit, or Google Fit. Device name refers to the specific hardware or device label associated with the recorded observation. Date of first device linkage indicates the date on which a given device was first connected to the health record. Measurement type indicates the category of health data captured, such as step count or pulse. Measurement value refers to the observed numeric value for that measurement, and date of observation indicates the calendar date on which the measurement was recorded.

Further limitations

A limitation that should be noted beyond those in the discussion is our operational definition of disengagement as no further valid EHR-linked wearable observations after a patient's last recorded observation. This endpoint is agnostic to cause and therefore may reflect true behavioral disengagement, such as opting out or stopping use, but also nonbehavioral or involuntary factors such as device breakage, device replacement or switching without re-linking, app permission changes, synchronization failures, or other technical disruptions. In addition, consumer device ecosystems differ in how data are routed through manufacturer-specific or third-party apps or platform APIs to the EHR, and these pathways may not be equally reliable across platforms. As a result, apparent disengagement may sometimes reflect a change in device, app, or data-routing configuration rather than cessation of self-monitoring. Accordingly, the outcome should be interpreted as cessation of observable EHR-linked data transmission, not definitive cessation of wearable use, and comparisons of time-to-disengagement across device types should be interpreted cautiously.

2.0 Supplementary Figures

Supplementary Figure 1: Overall Survival

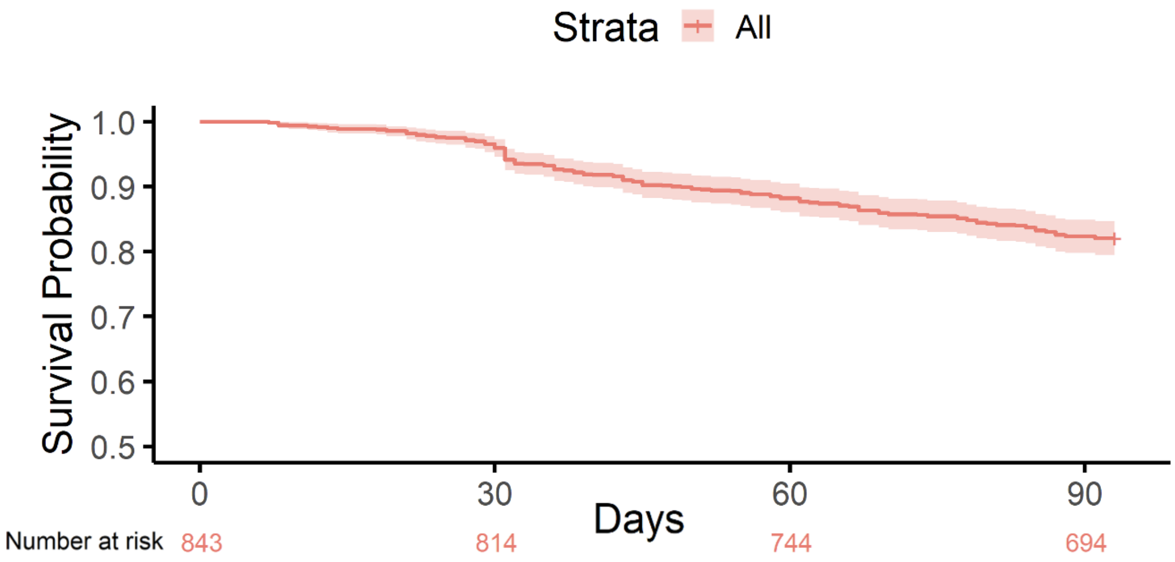

Supplement: Supplementary file 1 [file DataSheet1.pdf]
